# Root Exudates Are Linked to Antibiotic Resistance Gene Variation by Modulating Rhizosphere Microbial Community Assembly Under Swine Wastewater Irrigation

**DOI:** 10.3390/antibiotics15050444

**Published:** 2026-04-29

**Authors:** Liwei Liu, Meng Wang, Xiuzhi Wang, Yuan Liu, Zhongyang Li

**Affiliations:** 1 Institute of Farmland Irrigation of CAAS, Xinxiang 453002, China; llw961130@163.com (L.L.); wangmeng231120@163.com (M.W.); wxz3308959@163.com (X.W.); 2 Xinxiang Agricultural Water and Soil Environmental Field Science Research Station, Chinese Academy of Agricultural Sciences, Xinxiang 453002, China; 3 National Observation and Research Station of Shangqiu Agro-Ecology System, Shangqiu 476000, China

**Keywords:** ARGs, microecological regulation, rhizosphere microorganisms, root exudates, swine wastewater

## Abstract

**Background**: Irrigation with swine wastewater may increase the dissemination risk of antibiotic resistance genes (ARGs) in the rhizosphere and alter root exudate composition. However, the relationship between root exudates and ARG dynamics under swine wastewater irrigation remains poorly understood. This study therefore aimed to clarify how root exudates are connected with ARG dynamics under swine wastewater irrigation. **Methods**: To address this, untargeted metabolomics and metagenomic sequencing were combined to characterize rhizosphere ARG composition, microbial community structure, and root exudate profiles in different soybean cultivars under swine wastewater irrigation. **Results**: The results showed that irrigation water source and soybean cultivar were associated with variation in soil ARG composition and changes in plant root metabolic profiles. Under wastewater irrigation, the relative abundances of secondary metabolites in root exudates were generally elevated, particularly those of organic nitrogen compounds and organic oxygenated compounds. Cultivar-related variation remained evident in rhizosphere microbial communities and ARG profiles, and differences in exudate composition among cultivars became smaller. Irrigation water source and soybean cultivar were associated with changes in ARG dynamics. This association was mainly linked to variation in rhizosphere microbial community structure rather than direct effects of root exudates on ARGs. Xanthine and 3-isobutylpentanedioic acid, identified as key root exudates, increased under wastewater irrigation and were related to variation in the potential ARG host genus SCGC-AG-212-J23 and the related ARGs. In contrast, 5-methylheptan-3-one decreased under wastewater irrigation and was correlated with variation in SCGC-AG-212-J23, Gp6-AA40, and the related ARGs. **Conclusions**: Swine wastewater irrigation and soybean cultivar altered root metabolism, which were linked to variation in rhizosphere microbial communities. These changes may have collectively contributed to shifts in rhizosphere ARGs. This could provide a basis for understanding the ecological relationships among root exudates, microorganisms, and ARGs under swine wastewater irrigation.

## 1. Introduction

Against the backdrop of increasing pressure on agricultural resources, swine wastewater, as a typical recycled agricultural water source, has been increasingly used in agricultural production [1,2,3]. Swine wastewater is rich in nutrients such as nitrogen, phosphorus, and potassium [4,5,6]. It can supplement soil nutrients, improve soil fertility, and reduce reliance on chemical fertilizers [7,8]. However, swine wastewater also contains residual antibiotics, antibiotic-resistant bacteria, and antibiotic resistance genes (ARGs), and its long-term application can lead to the accumulation of ARGs in soil [9,10,11,12,13]. In addition to proliferation through microbial growth, ARGs may also be transferred through horizontal gene transfer (HGT) [14,15]. They can further infiltrate agricultural microbial systems and even the food chain, thereby posing potential risks to ecological security and human health [16,17,18]. Swine wastewater irrigation can exert direct effects on the plant rhizosphere [19]. As a key ecological interface for the migration of exogenous substances and the assembly of microbial communities, the rhizosphere has received increasing attention [20,21,22]. Among rhizosphere factors, root exudates play a central role. Comprising organic acids, sugars, phenolic compounds, and various secondary metabolites, they serve as carbon sources for microorganisms and also shape microbial community assembly through signaling functions [23,24,25]. The composition and release of root exudates are strongly influenced by plant species, rhizosphere conditions, and external environmental stress. Under drought, nutrient limitation, or symbiotic activation, plants can reshape the rhizosphere microenvironment by adjusting exudate composition [26]. During this regulatory process, plant root exudates influence microbial growth and metabolic activities [27,28]. They also alter microbial community structure [29,30]. These changes may further affect the environmental expression and dissemination of antibiotic resistance genes [31,32].

Previous studies have suggested that root exudates may regulate ARGs through their effects on rhizosphere microorganisms. For example, in the tomato rhizosphere, the specific metabolite luteolin-7-glucoside reduced the abundance of *tetA(58)* [33]. Root exudates with biocontrol functions are reported to exhibit strong correlations with most ARGs. Vegetable species also significantly influence the abundance of soil ARGs [34]. Sinapyl alcohol and 6-gingerol secreted by ginger roots can enhance immune activation and the colonization of beneficial functional microorganisms, thereby reducing the expression of plant Fusarium wilt resistance genes [35]. Similar mechanisms have also been observed in potato onion (*Allium cepa* var. agrogatum). Taxifolin released from potato onion roots can regulate the root metabolism of neighboring tomato plants, recruit beneficial Bacillus species, and protect tomato plants from Verticillium wilt caused by the soil-borne pathogen Verticillium dahliae [36]. In other words, plants can mediate the restructuring of microbial communities through metabolic regulation, thereby intervening in the environmental behavior of ARGs [37]. From the perspective of plant disease resistance, these studies have demonstrated that root exudates shape microbial community structure and influence the distribution of disease-resistance genes. These findings also suggest that root exudates may have the potential to regulate the distribution of ARGs. However, direct evidence for the effects of root exudates on ARGs remains limited. In addition, most existing studies have been conducted under freshwater irrigation, whereas the role of irrigation water quality in these processes has received much less attention. In particular, under swine wastewater irrigation, whether a coupled relationship exists among root exudates, rhizosphere microorganisms, and ARGs remains unclear. Previous studies have shown that crop type and species, as well as external stressors, can induce dynamic changes in root exudate composition, rhizosphere microbial community structure, and ARGs during crop growth. Thus, we hypothesized that both crop cultivar and irrigation water quality can affect root exudate composition, and that their interaction may reshape exudate profiles. These changes may further influence the colonization and functions of key microbial taxa and thereby the variation in ARG composition.

As an important source of edible oil and protein feed, soybeans play a critical role in global food security. Therefore, different soybean cultivars under swine wastewater irrigation were selected as the study system. Untargeted metabolomics combined with metagenomic sequencing was used to analyze root exudate composition, rhizosphere microbial community structure, and ARG composition. The aim of this study was to clarify the relationships among root exudates, microorganisms, and ARGs in the soybean rhizosphere under swine wastewater irrigation. The research findings can support the understanding and management of antibiotic resistance risks arising from the agricultural reuse of livestock wastewater.

## 2. Results

### 2.1. Soil Basic Properties

**Figure 1:**
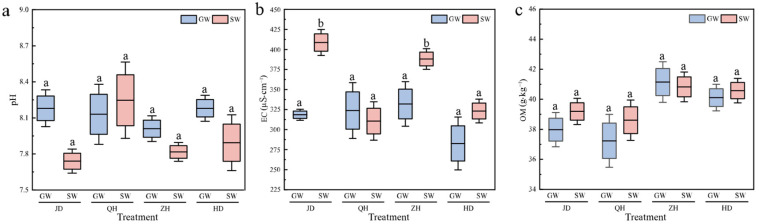
Rhizosphere soil pH (**a**), electrical conductivity (EC) (**b**), and organic matter (OM) content (**c**) of soybean cultivars JD, QH, ZH, and HD. Different lower case letters represent significant difference between treatments at *p* < 0.05. GW, groundwater; SW, swine wastewater; JD, Jidou 12; QH, Qihuang 34; ZH, Zhonghuang 13; HD, Hedou 33.

The basic properties of the rhizosphere at the soybean flowering-pod-setting stage are presented in Figure 1. The soil pH values range from 7.74 to 8.25, showing a decrease compared with the pre-planting value of 8.33. SW irrigation generally lowered soil pH, except for cultivar QH, which exhibited a slightly higher pH under SW irrigation than under GW irrigation. The patterns of electrical conductivity (EC) corresponded to the intergroup differences observed for pH. Higher EC values were observed under SW irrigation than under GW irrigation except for cultivar QH, and the post-planting EC values (282.71–408.66 μS·cm^−1^) were markedly higher than the pre-planting value of 122.80 μS·cm^−1^. Soil organic matter (OM) accumulated continuously during cultivation. For soils planted with cultivar ZH, OM content was higher under GW irrigation than under SW irrigation. For cultivars JD, QH, and HD, soil OM under SW irrigation increased by 3.20%, 3.68%, and 1.17%, respectively, compared with GW irrigation. As shown in Table A2, the irrigation water source had a significant effect on rhizosphere pH, whereas neither soybean cultivar nor its interaction with water source did. EC was significantly influenced by cultivar and irrigation water source, as well as their interaction. In contrast, soil OM responded significantly only to the soybean cultivar.

### 2.2. Crop Growth Traits

**Figure 2:**
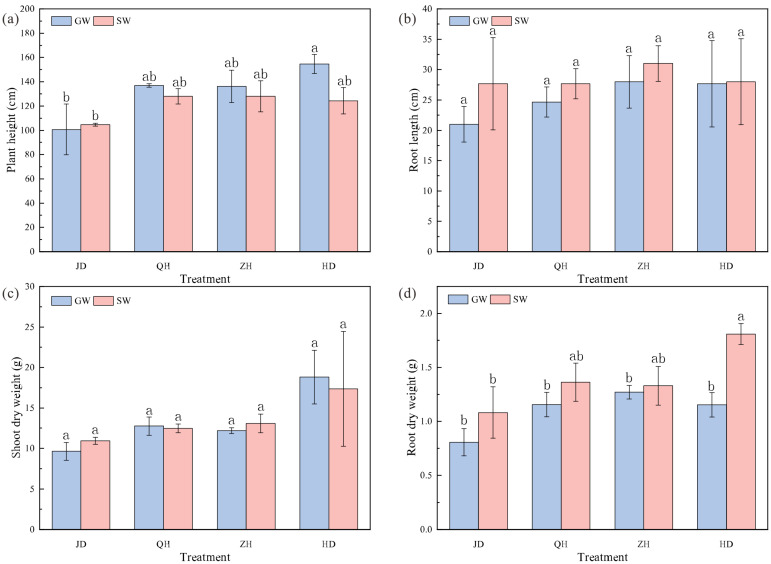
Plant height (**a**), root length (**b**), shoot dry weight (**c**), and root dry weight (**d**) of soybean cultivars JD, QH, ZH, and HD under groundwater (GW) and swine wastewater (SW) irrigation. Different lower case letters represent significant difference between treatments at *p* < 0.05. JD, Jidou 12; QH, Qihuang 34; ZH, Zhonghuang 13; HD, Hedou 33.

The growth performance of soybean plants at the flowering-pod-setting stage is shown in Figure 2. Compared with GW irrigation, SW irrigation exerted pronounced effects on plant growth. SW irrigation increased the root dry weight, but this increase was significant only for cultivar HD. Root length did not show a significant response to irrigation water sources. For aboveground traits, cultivar-specific responses to SW irrigation were observed. SW irrigation increased plant height and shoot dry weight of JD and reduced those of HD, but neither change was significant. Some cultivars showed greater plant height under GW irrigation. However, their shoot dry weight did not increase correspondingly. This suggests no direct causal relationship between plant height and dry matter accumulation. Overall, soybean growth responses to SW irrigation were cultivar dependent, with variation mainly observed in root dry weight, plant height, and shoot dry weight.

### 2.3. Composition of Root Exudates

**Figure 3:**
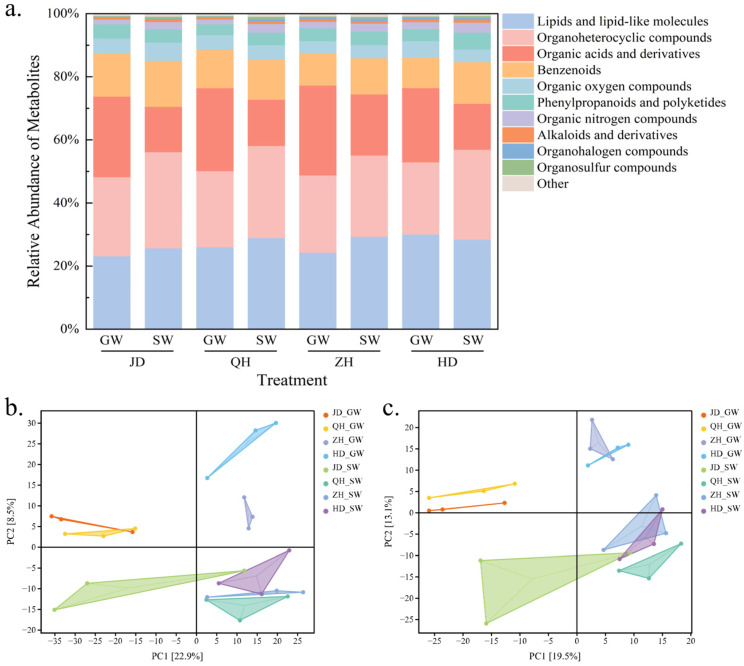
Composition and differential analysis of root exudates. (**a**) Relative abundances of the top 10 classes of root exudates from soybean cultivars JD, QH, ZH, and HD under groundwater (GW) and swine wastewater (SW) irrigation. Partial least squares discriminant analysis illustrating intergroup differences in the positive ion mode (**b**) and negative ion mode (**c**). JD, Jidou 12; QH, Qihuang 34; ZH, Zhonghuang 13; HD, Hedou 33.

By matching against an in-house database and public databases, a total of 3980 metabolites were identified, of which 1947 were successfully annotated in the HMDB database. Based on molecular structure, organic acids and their derivatives (14.4–28.5%), organic heterocyclic compounds (22.9–30.5%), and lipids and lipid-like molecules (23.3–30.2%) constituted the fundamental compositional framework of the rhizosphere metabolite profile (Figure 3a). In addition, phenylpropanoids and polyketides, organic oxygenated compounds, benzenoid compounds, and organic nitrogen compounds were also widely present at low to moderate abundances. These compounds reflected the diverse secondary metabolic responses of plant roots to environmental stimuli. Compared with GW irrigation, the relative abundances of secondary metabolites such as organic nitrogen compounds, phenylpropanoids, and polyketides, and organic oxygenated compounds were generally higher under SW irrigation. This suggests that swine wastewater irrigation may stimulate the release of signaling molecules from plant roots and rhizosphere microorganisms and enhance plant responsiveness to organic nitrogen and complex carbon sources. This trend was consistently observed across cultivars, suggesting that swine wastewater irrigation was associated with broad changes in the rhizosphere metabolic profile.

**Figure A1:**
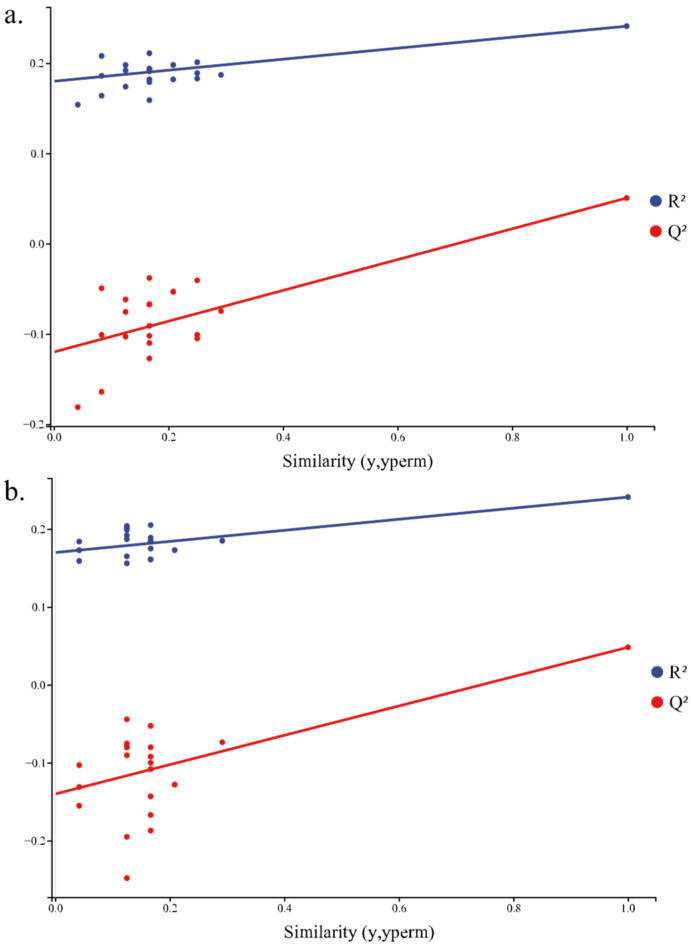
Permutation test of the PLS-DA model. Permutation tests of the PLS-DA models showed that the Q^2^ values in both POS (**a**) and NEG (**b**) modes were significantly higher than those obtained under random distributions, and that the intercepts of the Q^2^ regression lines were negative, confirming the discriminative stability and statistical robustness of the models.

PLS-DA revealed that both irrigation water source and cultivar jointly influenced the overall composition of soybean root metabolites (Figure 3b,c). In the positive (POS) and negative (NEG) ionization modes, PC1 and PC2 explained 31.4% and 32.6% of the total variance, respectively (Figure 3b,c). Root exudate profiles under GW and SW treatments were clearly separated along the PC2 axis, suggesting that the irrigation water source was an important factor contributing to metabolic differentiation. Root exudate composition varied more among cultivars under GW irrigation than under SW irrigation. In other words, cultivars varied in their sensitivity to water quality. In addition, permutation tests of the PLS-DA models (Figure A1) showed that Q^2^ values in both POS and NEG modes were markedly higher than those from random distributions. The intercepts of the Q^2^ regression lines were below zero, confirming the discriminative stability and statistical robustness of the models.

### 2.4. Differential Root Exudates and Metabolic Pathways

**Figure 4:**
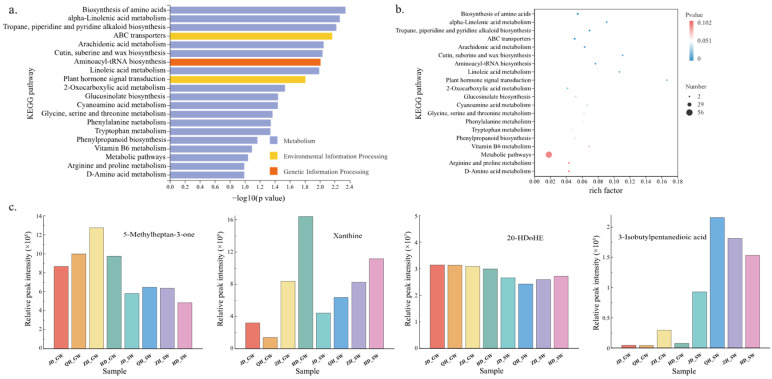
Enrichment analysis and abundance patterns of key differential root exudates. (**a**) Metabolic pathways of differential metabolites. (**b**) Pathway enrichment factors. (**c**) Relative abundance (RC, relative content) of key differential root exudates. GW, groundwater; SW, swine wastewater; JD, Jidou 12; QH, Qihuang 34; ZH, Zhonghuang 13; HD, Hedou 33.

Differential root exudates were significantly enriched in three major categories of metabolic pathways (Figure 4a,b), namely metabolism, environmental information processing, and genetic information processing. Among these, pathways associated with metabolism were dominant. They showed the greatest number and the most pronounced differences. This suggests that different treatments were associated with variation in plant metabolic pathways, particularly those related to carbon and nitrogen metabolism, lipid metabolism, and secondary metabolism. Pathways such as biosynthesis of amino acids, α-linolenic acid metabolism, and tropane, piperidine, and pyridine alkaloid biosynthesis showed a high level of enrichment. Together, these pathways constitute the metabolic foundation underlying the production and regulation of root exudates.

**Figure A2:**
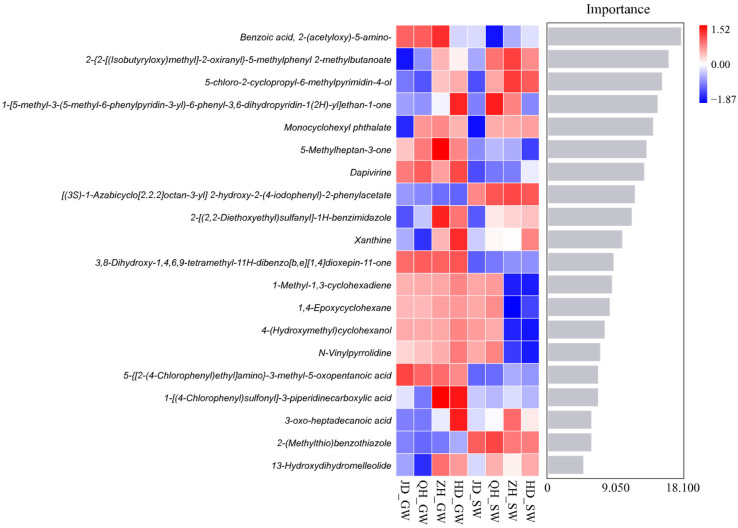
The top 20 metabolites ranked by importance scores identified using the random forest model across different groups. Metabolites are ordered from top to bottom by decreasing importance to the model; those with higher importance scores can be regarded as marker metabolites distinguishing among groups. GW, groundwater; SW, swine wastewater; JD, Jidou 12; QH, Qihuang 34; ZH, Zhonghuang 13; HD, Hedou 33.

Based on the PLS-DA results, the random forest model (Figure A2), and metabolic pathway analysis, four key differential root exudates were ultimately identified: 3-isobutylpentanedioic acid, xanthine, 5-methylheptan-3-one, and 20-HDoHE. These metabolites belong to organic acids, organic nitrogen compounds, organic oxygenated compounds, and lipids, respectively (Table A3). These results suggest that variation in root exudate composition may be associated with changes in the rhizosphere chemical environment under different irrigation water sources. These four key differential root exudates exhibited distinct distribution patterns across cultivars and irrigation treatments (Figure 4c). Among these, 3-isobutylpentanedioic acid showed a significantly higher abundance under SW irrigation than under GW irrigation, while the opposite was true for the volatile ketone 5-methylheptan-3-one. This suggests that differences in metabolite distribution may be associated with irrigation conditions. 20-HDoHE is an oxidized lipid signaling molecule whose abundance was comparable across treatments, implying that it was relatively insensitive to external conditions. Xanthine originates from amino acid biosynthesis and its derivative pathways, which provide key intermediates for nitrogen and energy metabolism and form the biochemical basis for purine compound synthesis. The distribution of xanthine was jointly influenced by cultivar and irrigation water source, with higher abundance in root exudates of cultivar HD than in other cultivars, and higher levels under GW irrigation than under SW irrigation. These results indicate that xanthine accumulation exhibited pronounced genotype specificity and was also sensitive to environmental conditions.

### 2.5. Soil Microbial Community Structure

**Figure 5:**
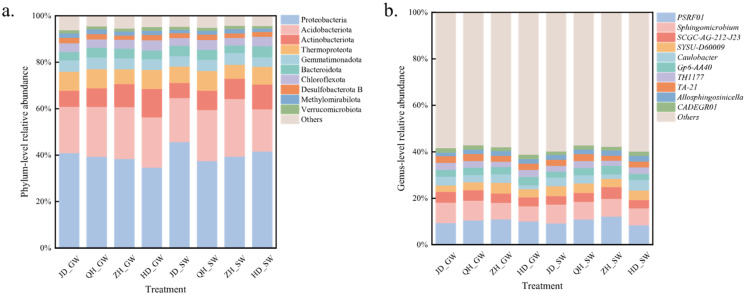
Composition of soil microbial communities. (**a**) Relative abundances of microbial phyla. (**b**) Relative abundances of microbial genera. GW, groundwater; SW, swine wastewater; JD, Jidou 12; QH, Qihuang 34; ZH, Zhonghuang 13; HD, Hedou 33.

The dominant bacterial phyla in the soil were *Proteobacteria*, *Acidobacteriota*, *Actinobacteriota*, *Gemmatimonadota*, and *Bacteroidota*, collectively accounting for more than 85% of the total community (Figure 5a). However, their relative abundances varied among treatments. Among these phyla, Proteobacteria and Acidobacteriota were the most abundant. Across the four cultivars, SW irrigation tended to enrich Proteobacteria and reduce Acidobacteriota compared with GW irrigation. Under GW irrigation, the relative abundance of Proteobacteria in the JD treatment (41.08%) was higher than in other treatments (34.86–39.46%), whereas the relative abundance of Actinobacteriota (6.90%) was lower than in other treatments (7.99–12.23%). A similar pattern was observed under SW irrigation. Results of PERMANOVA (Table A4) indicated that soybean cultivar significantly affected microbial community composition at both the phylum and genus levels. By comparison, the irrigation water source and its interaction with cultivar showed no significant effects. Dominant bacterial genera in the soil included *PSRF01*, *Sphingomicrobium*, *SCGC-AG-212-J23*, *SYSU-D60009*, and *Caulobacter*.

### 2.6. Soil Microbial Diversity

**Figure 6:**
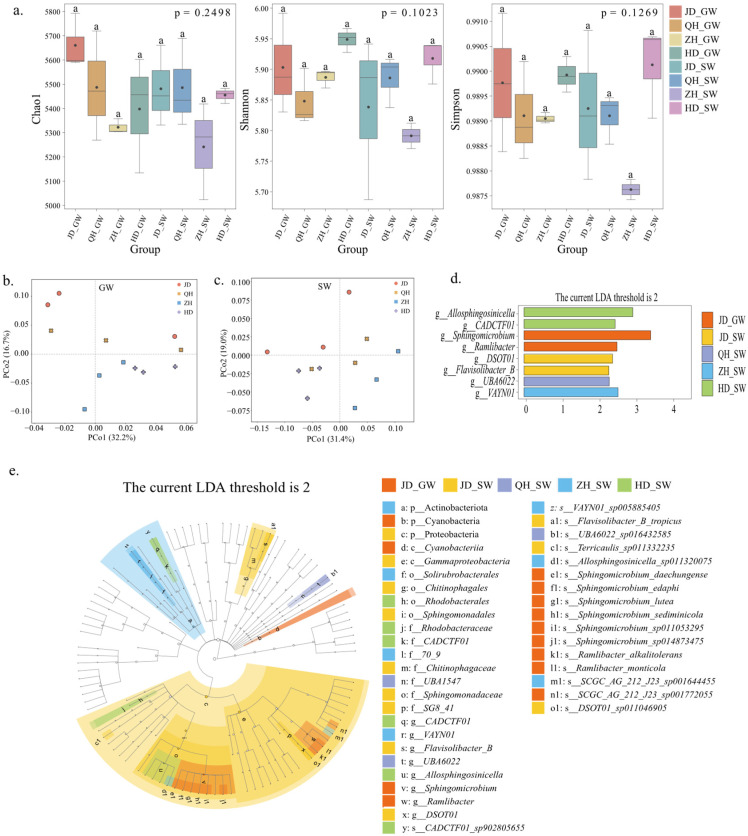
Analysis of differences in soil microbial community structure under different treatments. (**a**) Soil microbial α-diversity indices under different treatments. Different lower case letters represent significant difference between treatments at *p* < 0.05. (**b**) PCoA of soil microbial community composition under GW irrigation. (**c**) PCoA of soil microbial community composition under SW irrigation. (**d**) Genus-level LDA score plot of differentially abundant taxa identified by LEfSe. (**e**) LEfSe cladogram of differentially abundant taxa from the phylum to genus levels. GW, groundwater irrigation; SW, swine wastewater irrigation. GW, groundwater; SW, swine wastewater; JD, Jidou 12; QH, Qihuang 34; ZH, Zhonghuang 13; HD, Hedou 33.

We used three commonly applied indices, Chao1, Shannon, and Simpson, to characterize the α-diversity of soil microbial communities (Figure 6a). Neither irrigation water source nor cultivar had significant effects on them (*p* > 0.05), indicating that no significant differences in the overall richness and evenness of rhizosphere microbial communities were detected across treatments. Based on the PCoA results (Figure 6b,c), the first two principal coordinates explained 32.2% and 16.7% of the community variation under GW irrigation, compared to 31.4% and 19.0% under SW irrigation. Samples from the JD and QH treatments were more widely dispersed, whereas those from the ZH and HD treatments exhibited higher similarity. Compared with GW irrigation, samples under SW irrigation showed lower dispersion among the three replicates for each cultivar, suggesting reduced variability in community structure among replicates.

LEfSe analysis identified microbial taxa that were significantly enriched under specific treatments across multiple taxonomic levels, including phylum, class, order, family, genus, and species (Figure 6d,e). Overall, taxa showing significant abundance differences were more frequently detected under SW irrigation than under GW irrigation. Under SW irrigation, QH and ZH significantly enriched UBA6022 and VAYN01, respectively, while HD showed enrichment of *CADCTF01* and *Allosphingosinicella*. The LDA score plots further quantified the contribution of significantly differential taxa in each treatment. For cultivar JD, the enriched differential microbial groups differed between irrigation water sources. Under SW irrigation, *Flavisolibacter* and *DSOT01* were mainly enriched, along with an overall enrichment of *Sphingomonadaceae* at the family level. Under GW irrigation, *Sphingomicrobium* and *Ramlibacter* were mainly enriched, and the enrichment was mainly reflected at finer taxonomic levels, including *Sphingomicrobium* and its related species. In contrast to JD, the QH, ZH, and HD treatments exhibited fewer significantly differential taxa with lower LDA scores.

### 2.7. Composition and Diversity of ARGs

**Figure 7:**
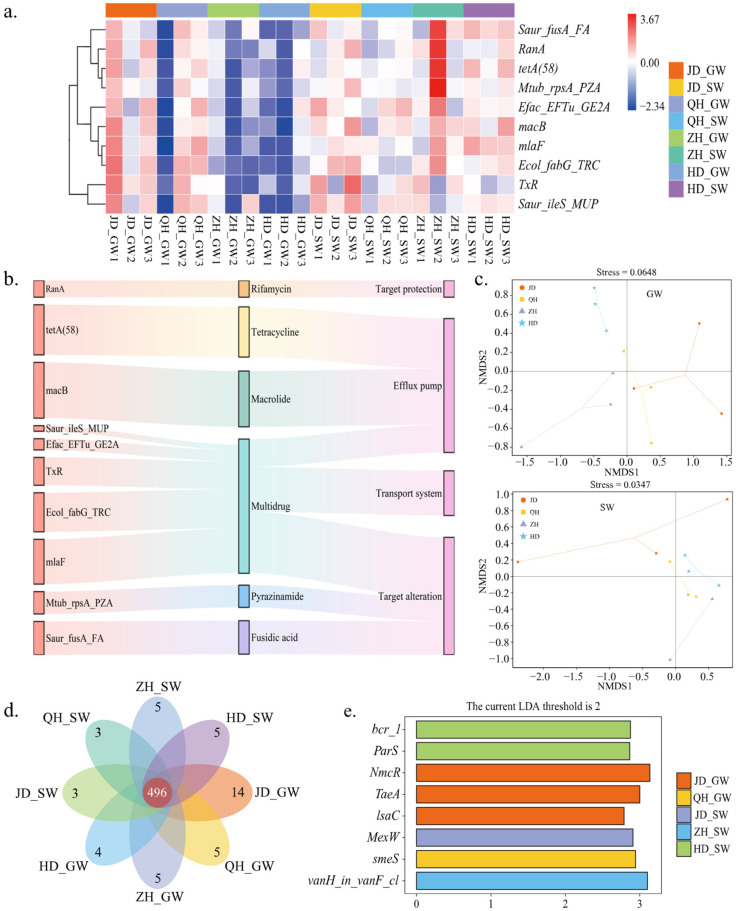
Composition and differential analysis of ARGs in soil under different treatments. (**a**) Heatmap of the 10 most abundant ARG subtypes. (**b**) Sankey diagram of ARG subtypes, antibiotic classes, and resistance mechanisms. (**c**) NMDS of soil ARG composition under different soybean cultivars and irrigation water sources. (**d**) Venn diagram of shared and unique ARG subtypes among different groups. (**e**) Linear discriminant analysis (LDA) score plot of differentially abundant ARGs identified by LDA Effect Size (LEfSe) analysis. GW, groundwater irrigation; SW, swine wastewater irrigation; JD, Jidou 12; QH, Qihuang 34; ZH, Zhonghuang 13; HD, Hedou 33.

Abundance profiles were constructed using the top 10 most abundant soil ARGs, including *macB*, *tetA(58)*, *mlaF*, *TxR*, *Saur_rplA_FA*, and *Ecol_fabG_TRC* (Figure 7a). The overall abundance of ARGs in cultivars ZH and HD was higher under SW irrigation than under GW irrigation, whereas JD and QH exhibited relatively minor changes between the two waters. These patterns suggest that ARG composition was sensitive to soybean cultivars and irrigation water sources. The identified ARGs were mainly associated with resistance to macrolides, tetracyclines, β-lactams, aminoglycosides, and quinolones (Figure 7b). Among these, macrolide and tetracycline resistance genes accounted for the largest proportions of the ARG pool. In terms of resistance mechanisms, these ARGs predominantly conferred resistance via target protection and efflux pump systems.

Non-metric multidimensional scaling analysis of ARG composition showed some separation trends among treatments under GW and SW irrigation (Figure 7c). Under GW irrigation, the four cultivar treatments exhibited relatively large dispersion, suggesting cultivar-related variation in ARG composition. Under SW irrigation, samples from QH, ZH, and HD treatments clustered more closely, whereas the three replicates of JD showed greater dispersion. A total of 496 ARG subtypes were shared among all treatments (Figure 7d), while the number of treatment-specific ARG subtypes was relatively small. The JD treatment under GW irrigation contained 14 unique ARG subtypes, compared to 3–5 in the other treatments. Overall, fewer treatment-specific ARG types were detected in soils under SW irrigation, suggesting relatively greater similarity in ARG composition among SW treatments. Based on LEfSe analysis (LDA threshold > 2), a total of eight ARG subtypes with significant differences were identified (Figure 7e). LEfSe analysis showed treatment-specific enrichment of several ARG subtypes. Under GW irrigation, the JD treatment was enriched in *NmcR*, *TaeA*, and *lsaC*, whereas the QH treatment was enriched in *smeS*. Under SW irrigation, cultivar JD was enriched in *MexW*, cultivar ZH in *vanH_in_vanF_cl*, and cultivar HD in *bcr_1* and *ParS*. These differential ARGs were mainly associated with resistance mechanisms involving efflux pump systems, membrane protein transport, and cell wall modification (Table A5).

### 2.8. Associations Among Root Exudates, Microorganisms, and ARGs

**Figure 8:**
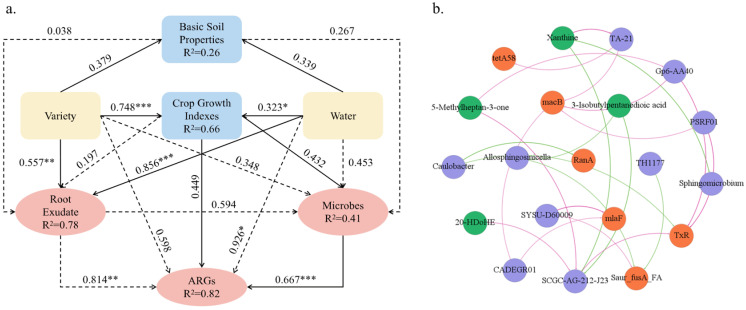
Relationships among root exudates, microorganisms, and ARGs. (**a**) Partial least squares path model (PLS-PM) of the direct and indirect effects of soil basic properties, soybean cultivar, crop growth indices, irrigation water source, root exudates, and microorganisms on soil ARGs. Numbers adjacent to the paths indicate path coefficients; solid lines represent direct effects, whereas dashed lines represent indirect effects. R^2^ values indicate the coefficients of determination of the variables in the model. * refers to *p* < 0.05, ** refers to *p* < 0.01, *** refers to *p* < 0.001. (**b**) Correlation network among root exudates, microorganisms, and ARGs. Green, purple, and orange nodes represent root exudates, microorganisms, and ARGs, respectively. Light green and pink edges indicate positive and negative correlations, respectively, at *p* < 0.05.

PLS-PM analysis results (Figure 8a) showed that variation in ARGs was more closely associated with the microbial community than with other variables. This was supported by a significant path coefficient from microorganisms to ARGs and a high explained variance of the ARG module. In contrast, root exudates did not show a significant direct association with ARGs. Instead, their relationship with ARGs appeared to be linked mainly through microbial variation. Similarly, the irrigation water source was not directly associated with ARGs. It may be linked to ARG variation through its associations with root exudate composition and microbial communities. A significant association was observed between soybean cultivar and root exudate composition.

**Figure A3:**
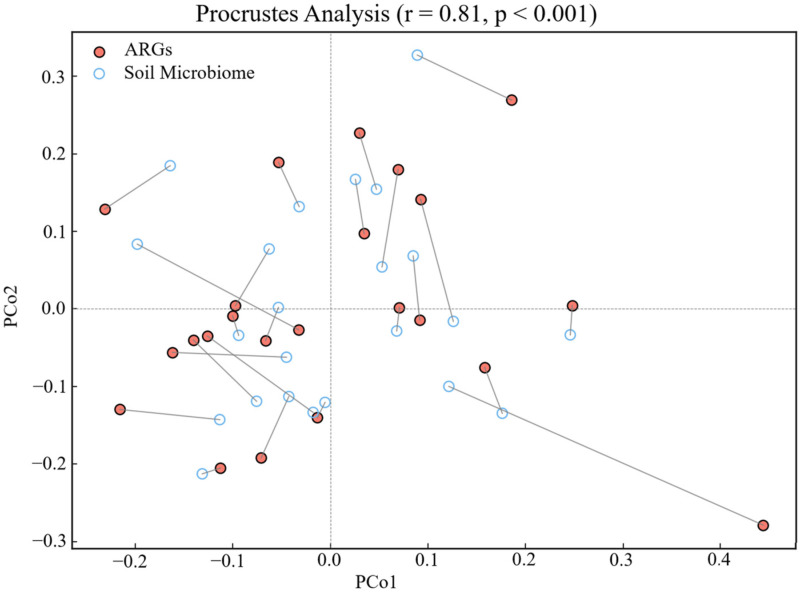
Procrustes analysis plot. Procrustes analysis indicated a strong coupling between microbial community structure and ARG profiles.

To further describe the relationships among root exudates, microorganisms, and ARGs, a correlation network was constructed. Network analysis results (Figure 8b) showed differences in the connectivity patterns of individual root exudates. Xanthine showed significant positive correlations with *TA-21* and significant negative correlations with Sphingomicrobium and *SCGC-AG-212-J23*, and these microbial nodes were also associated with major ARGs such as *TxR*, *macB*, and *tetA(58)*. In contrast, 5-methylheptan-3-one was predominantly positively correlated with *SCGC-AG-212-J23* and *Gp6-AA40*, which were also associated with ARGs such as *TxR* and *macB*. By comparison, 3-isobutylpentanedioic acid and 20-HDoHE exhibited relatively limited associations within the network, forming significant links with only a few microbial nodes and downstream ARGs. Overall, the correlation network displayed a hierarchical structure centered on microorganisms. Root exudates showed positive or negative associations with specific microbial taxa, which, in turn, were associated with ARGs. This network-level association pattern was consistent with the interaction pathways revealed by the PLS-PM analysis. Procrustes analysis further demonstrated a strong coupling between microbial community structure and ARG profiles (Figure A3; r = 0.81, *p* < 0.001).

## 3. Discussion

This study focused on rhizosphere ecosystem responses induced by irrigation water sources and systematically analyzed the coupled relationships among root exudate composition, microbial community structure, and the distribution of ARGs. The results suggest that soil ARGs are associated with plant metabolic characteristics and shifts in rhizosphere microbial community structure. This is consistent with previous findings highlighting the critical role of interactions between plants and microorganisms in regulating rhizosphere functions [38].

### 3.1. Joint Effects of Irrigation Water Source and Crop Cultivar on Rhizosphere System Dynamics

Irrigation water source and crop cultivar influenced different components of the rhizosphere system to different extents in our study. Irrigation water source was more closely associated with changes in soil physicochemical properties and root exudate composition, whereas microbial community composition was primarily influenced by soybean cultivar. This result is consistent with previous studies showing that soybean genotype regulated differences in rhizosphere microbial community structure [39]. Irrigation water source was associated with variation in rhizosphere soil pH and EC, while cultivar-specific differences and their interactions with irrigation water affected the magnitude of these changes. Compared with GW irrigation, SW irrigation lowered the rhizosphere pH of the weakly alkaline test soil. This differed from previous studies reporting that wastewater application increased soil pH in acidic soils [40], likely because the initial soil conditions were different. It may also be related to the increased release of acidic root exudates under SW irrigation in our study [41,42]. In terms of microbial responses, PERMANOVA indicated that variation in microbial community composition was primarily explained by soybean cultivar, with irrigation water source showing limited or non-significant effects. The results of PLS-PM further showed that irrigation water source, soybean cultivar, and root exudates did not directly act on ARG variation, but mainly affected ARGs indirectly through changes in microbial community structure. These findings suggest that the irrigation water source and crop cultivar played different roles in the rhizosphere system. The irrigation water source was more closely associated with environmental and metabolic variation, whereas the soybean cultivar was more strongly associated with microbial community composition. These microbiome shifts may also help explain the observed differences in crop growth traits under different irrigation treatments. Changes in rhizosphere community composition can affect nutrient turnover, root-associated interactions, and plant responses to environmental stress, thereby influencing plant growth performance. In the present study, SW irrigation was associated with cultivar-dependent variation in plant height, shoot dry weight, and root dry weight. These results suggest that microbial restructuring may be related to both ARG dynamics and plant growth responses. However, the direct contribution of microbial variation to crop growth was not explicitly tested in this study and requires further investigation.

### 3.2. Variation in Root Exudate Profiles Under Swine Wastewater Irrigation

As an important medium between plants and the rhizosphere environment, root exudates showed clear variation across different cultivars and irrigations, which is consistent with previous studies [43]. In this study, under SW irrigation, no uniform changes were observed across all metabolites. Notably, changes in the purine metabolism intermediate xanthine under SW irrigation exhibited pronounced cultivar dependence. Given the important role of purine metabolites in plant stress responses, this pattern may be related to differences in metabolic responses among genotypes under SW irrigation [44,45]. In contrast, the volatile medium-chain ketone 5-methylheptan-3-one, a potential signaling compound, generally decreased under SW irrigation. This change may reflect a plant strategy to suppress chemotactic signal transmission under complex microecological disturbances, thereby preventing excessive colonization by non-dominant microorganisms. In addition, 20-HDoHE, an oxidized derivative of docosahexaenoic acid, exhibited an overall decline under SW irrigation, suggesting that plants may mitigate exogenous oxidative stress by reducing lipid peroxidation levels [46].

### 3.3. Alterations in Root Exudates Recruit Adaptive Microorganisms

Previous studies have shown that host-derived compounds can regulate plant-associated microorganisms [47]. In our study, this relationship was reflected in shifts in rhizosphere microbial community structure associated with variation in root exudate composition. Under different irrigation conditions, shifts in root exudate composition provide distinct signals to microbial communities, thereby influencing the competitive status and colonization capacity of different microbial taxa within the community [16]. Previous studies have shown that certain low molecular weight metabolites can serve as readily utilizable substrates for microorganisms and as signaling molecules involved in microbial colonization and community assembly processes [48]. In this study, changes in root exudates did not occur synchronously across all compounds. Instead, fluctuations of key differential root exudates represented by xanthine and 5-methylheptan-3-one in the rhizosphere were associated with shifts in the relative abundances of several dominant or functionally important microbial taxa, such as *Sphingomicrobium*, *TA-21*, and *Gp6-AA40*. This suggests that these metabolites may be more closely associated with variation in microbial community composition. Overall, root exudates in the rhizosphere system may be associated with microbial community variation through their roles as carbon sources and as components of the rhizosphere chemical environment. The present results suggest that these associations were not limited to single microbial taxa but were reflected in broader changes in community composition. Microbial taxa that differed in their tolerance to environmental variation may therefore respond differently under changing rhizosphere conditions.

### 3.4. Associations Between Microbial Community Variation and ARGs Profiles

In this study, although the overall relative abundance of soil ARGs showed limited variation under different irrigation water sources, some variation in ARG composition and dominant types was observed among treatments. This pattern suggests that the differences in ARG profiles were not due to a widespread increase in resistance genes but were more likely associated with shifts in microbial community composition. Previous studies have also shown that microbial communities are important drivers of ARG diversity and distribution [49,50]. Our results further suggest that variation in ARG profiles in the rhizosphere may reflect corresponding changes in the microbial community. Considering the differential ARGs identified in this study, these genes were mainly associated with resistance mechanisms such as efflux pump systems, membrane protein transport, and cell wall modification. Such resistance mechanisms are typically linked to the basal adaptive capacity of microorganisms to environmental stress. Under SW irrigation, inputs of exogenous organic matter and dissolved nutrients likely altered rhizosphere resource conditions. These environmental changes may have influenced the ecological context of microbial and ARG responses. The shift in microbial community composition was more strongly associated with soybean cultivar than with irrigation water source. Similarly, PLS-PM suggested that irrigation water source and root exudates did not directly drive ARG variation, whereas microbial community variation did. The strong associations between multiple ARGs (*macB*, *tetA(58)*, and *TxR*) and specific microbial genera further support the coordinated responses of microorganisms and ARGs to environmental pressures. These associations may reflect responses of specific microbial genera and ARGs to changing environmental conditions, or coordinated variation in both microorganisms and ARGs under similar environmental selection pressures. Meanwhile, some ARGs (*Efac_EFTu_GE2A*, *Mtub_rpsA_PZA*, and *Ecol_fabG_TRC*) showed weak correlations with the most dominant genera, suggesting that their dynamics may exhibit strong species specificity. Given that variation in ARG profiles was more closely associated with microbial community variation, differences in microbial community composition may help explain the observed ARG patterns. In addition, interactions mediated by microorganisms may also affect broader rhizosphere functions, including processes related to plant growth, through nutrient cycling and interactions between plants and microorganisms [51,52].

### 3.5. Potential Mechanisms by Which Root Exudates Influence ARGs via Microorganisms

Different soybean cultivars and irrigation conditions directly changed root exudate composition and rhizosphere soil properties and indirectly altered microbial community composition. These changes may help explain the observed variation in ARG profiles. Root exudates were not directly associated with ARG profiles; instead, their relationship with ARGs appeared to be linked to microbial community composition and the distribution of potential host microorganisms. Under changing rhizosphere conditions, microbial taxa with different ecological tolerances may show distinct responses. Such variation may be associated with differences in the maintenance of corresponding ARGs within the community. In contrast, ARGs showing stronger host dependence may display more limited persistence during changes in community composition. Our findings suggest that root exudates may change the microbial environment around roots. This may alter microbial community structure and influence ARG profiles. Understanding these plant–microorganism interactions may provide a basis for managing ARG-related risks in agricultural systems.

### 3.6. Limitations and Future Perspectives

Several limitations should be considered when interpreting the findings of this study. The experiments were conducted in pots under controlled conditions, which facilitated the assessment of irrigation water source and cultivar effects, but may not fully represent the complexity of field environments. Thus, the general applicability of the observed patterns remains to be further evaluated under practical agricultural conditions. Due to the limited number of replicates causing high variability and the short experimental duration, we failed to observe a strong effect of root exudates on ARGs. In addition, the inferred relationships among root exudates, microbial communities, and ARGs were based mainly on correlation-driven analyses. These analyses support the identification of potential associations but, by themselves, do not establish causality.

Changes in ARG profiles in the microbiome associated with plant roots may also have implications for food safety and the potential transmission of resistance genes to consumers. In this study, the dominant ARGs, such as *macB* and *tetA(58)*, are associated with resistance to clinically important antibiotics, including macrolides and tetracyclines. Plant roots are an important interface for microbial colonization and exchange between soil and plant compartments. This allows ARG-carrying bacteria to persist in root-associated microbiomes. It also creates a possible route for their entry into agricultural production systems through root-associated materials or plant tissues. These ARGs, once present in the food system, could transfer resistance to the human gut microbiome or pathogens through HGT. However, since this study did not examine ARGs in plant tissues or their transfer to consumers, these implications remain speculative. Future studies should incorporate field validation, increased biological replication, and targeted experiments. These efforts will help clarify the ecological relationships among root exudates, microbial communities, and ARGs under swine wastewater irrigation.

## 4. Materials and Methods

### 4.1. Experimental Materials

Topsoil (0–20 cm) was collected from suburban farmland in Xinxiang City, air-dried, crushed, and passed through a 2 mm sieve prior to use. The basic soil properties were as follows: pH 8.33 (soil-to-water ratio of 1:5), electrical conductivity (EC) of 122.80 μS·cm^−1^ (soil-to-water ratio of 1:5), and organic matter (OM) content of 32.19 g·kg^−1^. The pot experiment was conducted in a vinyl tunnel at the Agricultural Water and Soil Environmental Field Science Observation Research Station of the Chinese Academy of Agricultural Sciences at Xinxiang. Groundwater (GW) and swine wastewater (SW) were used as irrigation water sources. Groundwater was collected from a depth of 4.5 m below the ground surface at the experimental station. Swine wastewater was obtained as digestate from an anaerobic fermentation tank of an intensive pig farm located near the experimental station after 30 days of fermentation. To ensure that the chemical oxygen demand (COD) of the swine wastewater met the requirements of the Standard for Irrigation Water Quality (GB 5084-2021) [52], the swine wastewater was diluted fivefold with groundwater. The basic properties of GW and the original SW are listed in Table A1.

### 4.2. Experimental Design

The pots used in the experiment measured 23 cm in top diameter, 19 cm in bottom diameter, and 21 cm in height. Each pot was filled with 7 kg of soil and amended with 0.7 g of compound fertilizer (N-P_2_O_5_-K_2_O ratio of 15:15:15; total nutrient content ≥ 45%). Four soybean cultivars widely cultivated in North China were selected for the experiment: Jidou 12 (JD), Qihuang 34 (QH), Zhonghuang 13 (ZH), and Hedou 33 (HD). For each cultivar, two irrigation treatments (GW and SW) were established, with three independent biological replicates per treatment. Sowing was carried out on 17 June 2024, and each pot was irrigated with 1 L of GW or SW according to the designated treatment. Two weeks after germination, seedlings were thinned to one plant per pot. Each pot was irrigated every 3–4 days with 0.5 L of GW or diluted SW according to the designated treatment, using a graduated plastic beaker to maintain soil moisture at 60–70% of field capacity. At the flower bud differentiation stage, urea (N ≥ 46%) was applied as a topdressing ferti-lizer at 75 kg·hm^−2^. The experiment concluded on 13 August 2024. The flowering-pod setting stage is a key period of soybean growth, during which plant metabolism is active, and root exudation is relatively abundant. Therefore, whole soybean plants were carefully uprooted at this stage, and soil samples shaken off from the roots were collected as rhizosphere samples. Soil samples from each treatment were divided into two subsamples: one was air-dried for the determination of soil basic properties, and the other was stored at −20 °C for subsequent DNA extraction. Soil pH and EC were measured in a 1:5 (*w*/*v*) soil-to-water suspension using a pH meter and a conductivity meter, respectively. Soil OM content was determined by the potassium dichromate oxidation-external heating method. After measuring soybean plant height and root length, the roots were gently rinsed with tap water and deionized water successively to remove attached soil. The plants were then transferred to conical flasks containing 0.5 L of deionized water (sufficient to fully immerse the root system) and incubated in the dark for 24 h. Subsequently, the collected root exudates were stored at −60 °C for subsequent analyses. After root exudate collection, the plants were dried in an oven at 65 °C to constant weight, and the shoot and root dry weights were recorded.

### 4.3. Untargeted Metabolomics Analysis

**Figure A4:**
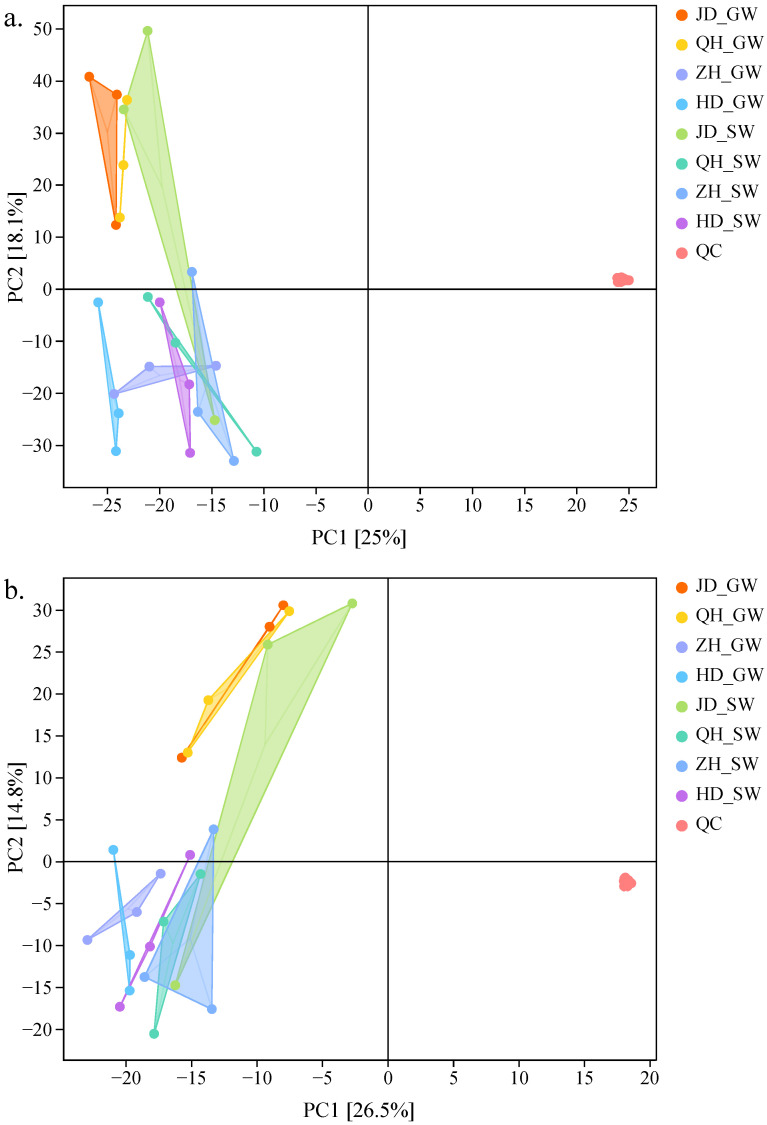
PCA score plots of metabolomic data including QC samples in positive (**a**) and negative (**b**) ion modes. GW, groundwater; SW, swine wastewater; JD, Jidou 12; QH, Qihuang 34; ZH, Zhonghuang 13; HD, Hedou 33.

Untargeted metabolomics analysis was carried out by Shanghai Personal Biotechnology Co., Ltd. (Shanghai, China). Prior to instrumental analysis, samples were extracted with pre-cooled methanol (Fisher, Waltham, MA, USA), followed by vortex mixing, tissue grinding, ultrasonication, and low-temperature incubation. The extracts were then centrifuged at high speed, and the supernatants were collected and filtered for metabolite detection. An aliquot of 100 μL from each root exudate sample was pooled to generate a quality control (QC) sample for analysis. An ultra-high-performance liquid chromatography (UHPLC) system (Vanquish, Thermo Fisher Scientific, Waltham, MA, USA) equipped with a Waters ACQUITY UPLC BEH Amide column (2.1 mm × 50 mm, 1.7 μm) (Milford, MA, USA) was used for chromatographic separation of the target compounds. Liquid chromatography was performed using an ACQUITY UPLC HSS T3 column (Waters, Milford, MA, USA, 100 A, 1.8 μm, 2.1 mm × 100 mm) at a flow rate of 0.4 mL min^−1^. The column temperature was maintained at 40 °C, the autosampler temperature was set to 8 °C, and the injection volume was 2 μL. Mass spectrometric data acquisition in both positive and negative ion modes was performed in data-dependent acquisition (DDA) mode using Xcalibur 4.4 software on Orbitrap Exploris 120 mass spectrometer (Thermo Fisher Scientific GmbH; Dreieich, Germany). A heated electrospray ionization (HESI) source was employed with spray voltages set at 3.5 kV (positive) and −3.0 kV (negative). The sheath gas and auxiliary gas were set to 40 and 10 arb, respectively. The capillary temperature was maintained at 320 °C, and the auxiliary gas heater temperature was set to 300 °C. Full MS scans were acquired at a resolution of 60,000 over an *m*/*z* range of 70–1000, with the AGC target set to Standard and a maximum injection time (Max IT) of 100 ms. The top four most intense precursor ions were selected for MS/MS fragmentation with a dynamic exclusion time of 4 s. MS/MS spectra were acquired at a resolution of 15,000 using higher-energy collisional dissociation (HCD) with a normalized collision energy of 30%, with the AGC target set to Standard and Max IT set to Auto. All formal samples and QC samples were analyzed using the chromatographic and mass spectrometric conditions described above. Prior to study sample injection, 2–4 QC injections were performed to equilibrate the analytical system. After data acquisition, peak extraction, alignment, filtering, and metabolite identification were conducted. The consistent clustering of QC samples in the PCA score plot (Figure A4) indicated acceptable analytical stability during metabolomic analysis. Root exudates were annotated using public databases, including mzCloud, LIPID MAPS, HMDB, MoNA, and NIST_2020_MSMS, as well as an in-house compound library. Metabolite identification was based on matching retention time, accurate mass (mass error < 10 ppm), MS/MS fragmentation spectra, and collision energy, followed by manual verification. The confidence level of metabolite identification was classified as MSI level 2 or higher.

### 4.4. Soil DNA Extraction and Metagenomic Sequencing

Total microbial genomic DNA was extracted from soil samples using the MagBeads FastDNA Kit (116564384; MP Biomedicals, Santa Ana, CA, USA). The quantity and quality of the extracted DNA were assessed using a Qubit 4 fluorometer (Thermo Fisher Scientific, Waltham, MA, USA). Sequencing libraries with an insert size of approximately 400 bp were prepared using the KAPA Hyper Prep Kit (Roche Sequencing Solutions, Pleasanton, CA, USA). Metagenomic sequencing was performed on the BGI-T7 high-throughput sequencing platform using a whole-genome shotgun (WGS) strategy by Shanghai Personal Biotechnology Co., Ltd. (Shanghai, China). Paired-end (PE) sequencing was conducted for all libraries. The sequencing output ranged from 36.26 to 56.12 million reads per sample, corresponding to 5.44–8.42 Gb of data, with an average of 45.51 million reads and 6.83 Gb per sample. The resulting reads were quality corrected using BBCMS and assembled into contigs using MEGAHIT (v1.2.9). Taxonomic annotation was then performed with Kraken2 against a customized database integrating bacterial genomes from GTDB, eukaryotic microbial sequences from NCBI-nt, and viral genome sequences from RVDB. ARGs were annotated against the Comprehensive Antibiotic Resistance Database (CARD) using MMseqs2 with a sensitivity of 5.7, a maximum sequence length of 100,000, and a search setting allowing up to 100 candidate matches for each query sequence. For each query sequence, only the top-scoring match was retained for annotation. To reduce the risk of false-positive annotations, ARG annotations were further filtered using a minimum sequence identity threshold of 80%, a minimum alignment coverage threshold of 70%, and an E-value cutoff of 1 × 10^−5^. The data were analyzed using the free online platform Personalbio GenesCloud (https://www.genescloud.cn (accessed on 23 September 2025)).

### 4.5. Data Analysis

Raw data on soil basic properties and crop growth indices were organized in Excel 2024, and data visualization was mainly performed using Origin 2021 (OriginLab Corp., Northampton, MA, USA). Statistical analyses were conducted using IBM SPSS Statistics 26.0 (IBM Corp., Armonk, NY, USA). Data normality was first assessed using the Shapiro–Wilk test, followed by Levene’s test to evaluate homogeneity of variances among groups. Variables meeting the assumptions of normality and homoscedasticity were compared using one-way analysis of variance (one-way ANOVA), with the significance level set at *p* < 0.05. Data that did not satisfy normality assumptions were analyzed using the nonparametric Kruskal–Wallis rank-sum test.

Untargeted metabolomics data were normalized by peak area and Pareto-scaled prior to multivariate analyses. Principal component analysis (PCA) and partial least squares discriminant analysis (PLS-DA) were conducted to identify metabolic differences among groups. The robustness of the PLS-DA model was further evaluated using permutation tests, and model performance was assessed based on R^2^ and Q^2^ values. Variable importance in projection (VIP) scores was extracted from the PLS-DA model, and candidate differential root exudates were screened by combining VIP values with statistical significance from multiple-group comparisons (*p* < 0.05). *p*-values from multiple comparisons were adjusted using the Benjamini–Hochberg (BH) method to control the false discovery rate (FDR). In this study, VIP > 2.0 was used as a stricter criterion to screen key differential metabolites, with reference to previous metabolomics studies [53,54]. Compared with the commonly used VIP > 1.0 threshold, this stricter criterion was intended to narrow the candidate pool and identify fewer metabolites with stronger discriminatory ability and clearer relevance for subsequent analysis. This approach provided a more focused basis for subsequent mechanistic investigation and follow-up experiments. These root exudates were further screened against the top 20 most important root exudates identified by a random forest model, ultimately yielding a set of representative key differential root exudates.

Microbial α-diversity indices (Chao1, Shannon, and Simpson) were computed in R (v4.2.1) using the vegan and microbiome packages and visualized with boxplots. Microbial β-diversity was calculated based on Bray–Curtis distance matrices using the same R environment and packages, and community structure was visualized using principal coordinates analysis (PCoA). Based on the same distance matrix, two-way permutational multivariate analysis of variance (PERMANOVA) was performed using PAST 3 software to systematically evaluate the effects of plant cultivar, irrigation water source, and their interaction on microbial community structure. For ARG analysis, ARG subtype abundance was based on RC (read count) values obtained from CARD annotation results. The top 10 ARG subtypes in the heatmap were ranked by average RC values across samples, and row standardization was applied only for heatmap visualization. Non-metric multidimensional scaling (NMDS) and Linear discriminant analysis effect size (LEfSe) were conducted based on the ARG abundance matrix, with Bray–Curtis distance used for NMDS. LEfSe was used to identify differentially abundant microbial genera and ARG subtypes among treatments (LDA threshold = 2.0). Partial least squares path modeling (PLS-PM) was applied to evaluate the direct and indirect relationships among soil basic properties, soybean cultivar, crop growth indices, irrigation water source, root exudates, soil microorganisms, and soil ARGs. A Spearman correlation analysis was performed to construct the co-occurrence network among root exudates, microbial taxa, and ARGs. Only correlations with a correlation coefficient > 0.6 and a significance level of *p* < 0.05 were retained to ensure the reliability of the network. The resulting network was visualized using Gephi 0.10.1. Procrustes analysis was further used to assess concordance among metabolites, microorganisms, and ARGs.

## 5. Conclusions

Under pot experimental conditions, both the irrigation water source and soybean cultivar were associated with variation in root exudate profiles, rhizosphere microbial communities, and ARG composition, but the strength of these associations differed among components. Irrigation exerted effects on soil basic properties, root exudate composition, and microbial communities. Under swine wastewater irrigation, variation in root exudate composition was more pronounced than that under groundwater irrigation, especially in organic acids, organic nitrogen compounds, lipids, and other secondary metabolites. The relative abundance changes in key differential root exudates showed clear differences among cultivars, but under swine wastewater irrigation, cultivar-related differences in root exudate composition tended to decrease. Among these key metabolites, xanthine and 3-isobutylpentanedioic acid had higher abundances under SW irrigation. These metabolites corresponded to variation in the potential ARG host genus *SCGC-AG-212-J23* and the related ARG *TxR*. The metabolite 5-methylheptan-3-one had lower abundance under SW irrigation and corresponded to variation in the potential ARGs host genera *SCGC-AG-212-J23* and *Gp6-AA40*, as well as the related ARGs *TxR* and *macB*. No close direct correspondence was observed between variation in root exudates and ARGs. In contrast, their relationship was more closely linked to differences in rhizosphere microbial community structure. This study further indicates that, under swine wastewater irrigation, rhizosphere ARG distribution was associated not only with external irrigation inputs but also potentially with soybean cultivar and the related rhizosphere ecological processes. More attention should be given in future studies to understanding variation in ARGs in agricultural soils from the perspective of coordinated changes in plant metabolic responses and microbial communities.

## Data Availability

The original contributions presented in this study are included in the article. Further inquiries can be directed to the corresponding authors.

## Appendix A

**Table A1:** Basic properties of irrigation water sources.

Water	Total N/mg·L^−1^	Total P/mg·L^−1^	COD/mg·L^−1^
Groundwater	0.61	0.12	1.75
Swine Wastewater	37.32	15.03	443

**Table A2:** One-way analysis of variance of soil pH, electrical conductivity, and organic matter.

	pH		Electrical Conductivity		Organic Matter	
	F	*p*	F	*p*	F	*p*
Soybean cultivar	1.87	0.1760	7.69	0.0021	6.69	0.0039
Irrigation water source	5.19	0.0368	15.61	0.0011	1.49	0.2390
Interaction	1.77	0.1930	3.83	0.0305	0.49	0.6970

**Table A3:** Basic information on key differential metabolites.

ID	Compound	Formula	*m*/*z*	RT [min]	VIP	*p*_Value
MN3300	3-Isobutylpentanedioic acid	C_9_H_16_O_4_	169.087	4.385	2.232	1.65 × 10^−5^
MN2726	Xanthine	C_5_H_4_N_4_O_2_	151.026	0.907	2.175	4.69 × 10^−5^
MP3225	5-Methylheptan-3-one	C_8_H_16_O	129.128	5.406	2.135	5.52 × 10^−7^
MN10462	20-HDoHE	C_22_H_32_O_3_	325.218	6.446	2.184	2.24 × 10^−5^

**Table A4:** Two-way permutational multivariate analysis of variance of microbial community composition at the phylum and genus levels.

	Phylum Level		Genus Level	
	F	*p*	F	*p*
Soybean cultivar	5.14	0.01	2.84	0.01
Irrigation water source	3.01	0.06	0.34	0.84
Interaction	2.14	0.07	1.52	0.17

**Table A5:** Functions of differential ARGs.

ID	Antibiotic Class Resisted	Resistance Mechanism	Mechanism Category
*NmcR*	β-lactams	Transcriptional regulator associated with β-lactamase expression	Resistance regulation
*TaeA*	β-lactams	β-lactamase-mediated hydrolysis of the β-lactam ring	Antibiotic inactivation
*lsaC*	Macrolides, lincosamides, streptogramin A	ABC-F family ribosomal protection protein preventing antibiotic–ribosome binding	Target protection
*smeS*	Multiple antibiotic classes	RND family efflux pump component mediating antibiotic export	Efflux pump
*MexW*	Multiple antibiotic classes	RND family multidrug efflux pump protein	Efflux pump
*vanH_in_vanF_cl*	Glycopeptides	Modification of peptidoglycan precursors, reducing antibiotic binding	Target alteration
*bcr_1*	Peptide antibiotics	ABC transporter–mediated antibiotic efflux	Efflux pump
*ParS*	Multiple antibiotic classes	Two-component regulatory system involved in controlling membrane-associated resistance responses	Resistance regulation
